# Trends in incidence, mortality and survival in women with breast cancer from 1985 to 2012 in Granada, Spain: a population-based study

**DOI:** 10.1186/s12885-018-4682-1

**Published:** 2018-08-02

**Authors:** José Antonio Baeyens-Fernández, Elena Molina-Portillo, Marina Pollán, Miguel Rodríguez-Barranco, Rosario Del Moral, Lorenzo Arribas-Mir, Emilio Sánchez-Cantalejo Ramírez, María-José Sánchez

**Affiliations:** 1 Departamento de Urgencias y Emergencias, Área de Gestión Sanitaria Noreste, Hospital Regional de Baza, Carretera de Murcia s/n, 18800 Baza, Spain; 2 Escuela Andaluza de Salud Pública, Instituto de Investigación Biosanitaria ibs, Hospitales Universitarios de Granada/Universidad de Granada, Granada, Spain; 3 Public Health and Epidemiology CIBER Network (CIBERESP), Madrid, Spain; 4 Environmental and Cancer Epidemiology Department, National Center of Epidemiology - Instituto de Salud Carlos III, Madrid, Spain; 5 Department of Radiotherapy and Oncology, Virgen de las Nieves University Hospital, Granada, Spain; 6 Centro de Salud La Chana, Área de Gestión Sanitaria Granada-Metropolitano, Granada, Spain; 7 Department of Epidemiology and Public Health, University of Granada, Granada, Spain

**Keywords:** Cancer, Breast, Trend, Population-based, Incidence, Mortality, Survival, Stage, Registry, Spain

## Abstract

**Background:**

The incidence of breast cancer has increased since the 1970s. Despite favorable trends in prognosis, the role of changes in clinical practice and the introduction of screening remain controversial. We examined breast cancer trends to shed light on their determinants.

**Methods:**

Data were obtained for 8502 new cases of breast cancer in women between 1985 and 2012 from a population-based cancer registry in Granada (southern Spain), and for 2470 breast cancer deaths registered by the Andalusian Institute of Statistics. Joinpoint regression analyses of incidence and mortality rates were obtained. Observed and net survival rates were calculated for 1, 3 and 5 years. The results are reported here for overall survival and survival stratified by age group and tumor stage.

**Results:**

Overall, age-adjusted (European Standard Population) incidence rates increased from 48.0 cases × 100,000 women in 1985–1989 to 83.4 in 2008–2012, with an annual percentage change (APC) of 2.5% (95%CI, 2.1–2.9) for 1985–2012. The greatest increase was in women younger than 40 years (APC 3.5, 95%CI, 2.4–4.8). For 2000–2012 the incidence trend increased only for stage I tumors (APC 3.8, 95%CI, 1.9–5.8). Overall age-adjusted breast cancer mortality decreased (APC − 1, 95%CI, − 1.4 – − 0.5), as did mortality in the 50–69 year age group (APC − 1.3, 95%CI, − 2.2 – − 0.4). Age-standardized net survival increased from 67.5% at 5 years in 1985–1989 to 83.7% in 2010–2012. All age groups younger than 70 years showed a similar evolution. Five-year net survival rates were 96.6% for patients with tumors diagnosed in stage I, 88.2% for stage II, 62.5% for stage III and 23.3% for stage IV.

**Conclusions:**

Breast cancer incidence is increasing – a reflection of the evolution of risk factors and increasing diagnostic pressure. After screening was introduced, the incidence of stage I tumors increased, with no decrease in the incidence of more advanced stages. Reductions were seen for overall mortality and mortality in the 50–69 year age group, but no changes were found after screening implementation. Survival trends have evolved favorably except for the 70–84 year age group and for metastatic tumors.

## Background

Breast cancer is the most frequent tumor in women worldwide, particularly in countries with a high Human Development Index [1]. Moreover, it is one of the leading causes of cancer mortality in females. In 2015 there were 2.4 million estimated new cases and 523,000 estimated deaths worldwide in women, which correspond to about 29% of the total incident cancer cases and 14% of all cancer deaths [2]. There is huge variability in the incidence among countries, from 27 cases per 100,000 women in Asia to 97 per 100,000 white women living in the USA [3]. In Spain, the 2015 age-standardized incidence rate referred to the world population (ASR-W) was 65.2 per 100,000 women and the age-standardized incidence rate referred to the European population (ASR-E) was 88.3 per 100,000 women, placing this country in an intermediate position worldwide [4].

Several industrialized countries including Spain have shown a 30 to 40% increase in breast cancer incidence since the 1970s [3, 5]. This rise has been related to the spread of environmental and lifestyle risk factors, and to changes in diagnostic patterns [3, 6, 7]. A trend change has been observed since the beginning of the twenty-first century, mainly among women older than 50 years [8–10]. The main factors related to this change are the implementation of population-based screening programs at a country-wide level, and (albeit with a relatively low impact in Spain), the evolution of prescribing practices for hormonal replacement therapy [8, 11]. Analysis of breast cancer incidence trends in young women vary widely among countries, but in general show a steady increase since the early 1980s even in countries where the incidence in older age groups has decreased [12–14]. Studies in European countries and in the US show an increase in the incidence of early-stage tumors and a parallel reduction in late-stage tumors, although this reduction seems to be smaller than expected and the incidence of metastatic breast cancer has remained stable [15–18]. In Spain, there are no available population-based data on breast cancer incidence trends by stage.

Breast cancer mortality in Europe showed an increasing trend until the 1990s [3]. Between 1989 and 2006, breast cancer mortality (ASR-E) in European countries reportedly declined by a median of 19% [19]. The world-standardized mortality rate in Europe decreased from 21.3 in 1990 [20] to 16.7 deaths per 100,000 women in 2007 [21]. Finally, in Spain, the mortality rate (ASR-W) dropped from 17.3 per 100,000 women in 1995 to 10.8 per 100,000 in 2014 [22]. This reduction in mortality has been consistently smaller in women older than 70 years [5, 19], and correlates with the development of adjuvant treatments and, to a lesser extent, with the introduction of screening [23, 24].

Survival rates for breast cancer have generally increased since the 1980s. This trend has been related with a higher proportion of cases diagnosed at earlier stages as well as therapeutical improvements [25]. Currently, the 5-year net survival rate is higher than 85% in seventeen countries worldwide. In Europe the median survival rate ranges from 81 to 84%, with the exception of Eastern countries, where the survival rate is around 69% [26, 27]. However, no relevant increase in overall survival has been observed for metastatic tumors, or in the group of women older than 70 years [26, 28]. Spain had a 5-years survival rate of 78.4% for women diagnosed between 1997 and 1999, and this rate increased to 82.8% for those diagnosed between 2000 and 2007 [29]. Increasing trends in survival are related to early diagnosis and improvements in surgical and adjuvant treatments. Several recent studies have improved our understanding about the role played by screening, the spread of adjuvant treatments and their adverse effects, but there is still considerable controversy on this issue [30–32].

Since 1985 the Granada Cancer Registry (southern Spain) has systematically compiled data on breast cancer incidence, mortality, and crude and net survival trends. We were able to use the data collected for a period of more than 28 years from 1985 to 2012. In addition, we analyzed a subset of the data for the years 2000 to 2012, after the implementation of a screening program in 1998. For this period, we analyzed breast cancer incidence trends according to disease stage, to shed light on the impact of screening on stage distribution and its association to mortality and survival trends. To date no such analysis has been undertaken in Spain, as far as we are aware.

Determinants of breast cancer trends have been identified in previous studies, but unresolved controversies remain about their role. Trends studies provide an excellent opportunity to explore the specific weight of each factor. Studies at regional or national level frequently only consider either incidence or mortality [8, 15, 33, 34]. However, we present a comprehensive population-based analysis of breast cancer epidemiology, including every indicator and age group – an approach which facilitates an integral interpretation of the factors that may influence trends. Moreover, our analysis of tumor stages at diagnosis, together with the long observation period, hold the potential to provide a better understanding of trend determinants and especially the influence of breast cancer screening.

## Methods

### Participants and data sources

The population data were from the Granada Cancer Registry, a population-based cancer registry in southern Spain launched in 1985 and covering a population of about 922,100 inhabitants (50.3% women) (2011 population census of Granada. Source: Statistics and Cartography Institute of Andalusia http://www.juntadeandalucia.es/institutodeestadisticaycartografia).

A total of 8502 women residing in Granada province were diagnosed with a first primary invasive breast cancer, and 2470 breast cancer deaths were registered between January 1st 1985 and December 31st 2012. The Granada Cancer Registry uses as information sources public and private hospitals at the local and regional levels, oncology and pathology department records, and death certificates. Mortality data were extracted from the database of the Institute of Statistics and Cartography of Andalusia (http://www.juntadeandalucia.es/institutodeestadisticaycartografia). Other sources of information used, when necessary and available, were the National Index of Deaths (http://www.msssi.gob.es/estadEstudios/estadisticas/estadisticas/estMinisterio/IND_TipoDifusion.htm), the Social Security Database (http://www.seg-social.es/wps/portal/wss/internet/EstadisticasPresupuestosEstudios/Estadisticas), municipal census information, and hospital and primary care records.

The data in this registry are published regularly in Cancer Incidence in Five Continents (CIFC) monographs. The quality of the data is supported by good indicators: 96% of breast cancer cases were confirmed histologically, and a death certificate was the only source of information for 1.8% of the cases. Moreover, the Granada Cancer Registry is a member of the European Network of Cancer Registries (ENCR) and the Spanish Network of Cancer Registries (REDECAN), and a collaborator in the EUROCARE (http://www.eurocare.it/) and CONCORD studies (http://csg.lshtm.ac.uk/research/themes/concord-programme/).

### Study variables

Standard international procedures for cancer registries and coding rules are used in the Granada Cancer Registry. Breast cancer is defined as code C50 according to the International Statistical Classification of Diseases and Related Health Problems, 10th revision [35].

Age was stratified in 5-year intervals for standardization, and in the following broader groups for specific analysis: less than 40 years, 40–49, 50–69, 70–84, and 85 years or more. These groups have been established to focus on main topics concerning breast cancer trends, as has been done in previous analyses [13, 36–38]. Tumor stage at diagnosis was coded with the TNM system (6th edition for 2000–2010 and 7th edition for 2010–2012). Every case was re-coded according to the 7th edition [39].

Passive and active follow-up of cancer cases was carried out from the date of diagnosis to the end of follow-up (31 December 2014), when vital status was ascertained. The outcome variables were alive at the end of follow-up, death including date of exitus for any cause, or censored due to loss or incomplete follow-up.

### Statistical analysis

The number of new cases and deaths, crude rates, and age-standardized mortality and incidence rates referred to the European population are reported here. ASR-E rates were calculated by weighting age-specific incidence rates to the standard European population, and are expressed per 100,000 women-years. For incidence and mortality rates, R software was used (https://www.r-project.org).

Joinpoint regression analysis [40] of age-standardized or age-specific incidence or mortality rates was used to estimate the annual percentage change (APC) in breast cancer incidence and mortality. The APC was calculated by fitting connections between log scale linear trends to the chronological year as the regressor variable, assuming constant variance and uncorrelated errors. In the regression analysis, up to three change points (four trend line segments) were allowed. Each trend line segment is expressed by an APC value. When no change points were found, only one APC value represented the trend line for the whole period.

Joinpoint regression was performed on data from the earliest available data until the last year of available data. Stage at diagnosis was not systematically recorded in the Granada Cancer Registry for any cancer until the year 2000. Therefore, Joinpoint analysis of breast cancer incidence according to stage was only performed for the period 2000–2012.

Increasing or decreasing trends were considered to exist for *p* values < 0.05. The APC and 95% confidence intervals (CI) were calculated for the whole population, and for age groups (0–39 years, 40–49, 50–69, 70–84, and 85 years or more) and by tumor stage at diagnosis (2000 to 2012). For all statistical analyses we used the Joinpoint regression program (v. 4.1.1) [40].

Observed survival was calculated with the Kaplan-Meier method for 5-year periods from 1985 to 2009, and for the last 3-year period from 2010 to 2012. Because comorbidities can influence death rates, net survival was also calculated. This was defined as survival for cases in which breast cancer was the only cause of death. Net survival was estimated with the Pohar–Perme method [41] and cohort analysis. For 2010–2012, period analysis was used because follow-up time was too short for cohort analysis [42]. Survival (standardized and non-standardized by age) was calculated for 1, 3 and 5 years from diagnosis. Survival estimates were limited to ages 15–99 years, and we excluded cases for which a death certificate was the only source of information and those diagnosed on autopsy. Survival analysis was done with the strs package for Stata software v. 14 [43].

The dataset of the population-based cancer registry is registered as stipulated by law within the Spanish Data Protection Agency (Agencia Española de Protección de Datos. https://www.agpd.es). All data collected in the database for incidence, mortality and survival analysis were anonymous, and no ethical approval was required.

## Results

### Incidence 1985–2012

During the period from 1985 to 2012, 8502 new cases of breast cancer were registered among women living in Granada province (Table 1). Breast cancer accounted for 25% of all cancer cases (excluding non-melanoma skin cancer) in women during this time, and the median age at diagnosis was 59 years. European age-standardized incidence rates increased from 48 cases per 100,000 women in 1985**–**1989 to 83.4 per 100,000 women in 2008**–**2012 (Table 1), with a statistically significant APC of 2.5% (95%CI, 2.1–2.9).

**Table 1:** Breast cancer mortality and incidence rates, and numbers of cases and deaths, 1985–2012

Period	Incidence			Mortality		
	Cases	ASR-E*	ASR-W*	Deaths	ASR-E*	ASR-W*
1985**–**1989	909	48.0	36.0	408	20.5	14.8
1990**–**1994	1110	56.1	42.2	396	18.5	13.1
1995**–**1999	1363	64.1	47.7	420	17.9	12.6
2000**–**2004	1751	77.3	57.7	462	17.0	11.7
2005**–**2009	2051	81.0	60.5	485	16.5	11.6
2010**–**2012	1318	80.1	59.4	299	15.2	10.4
2000**–**2012	5120	79.5	59.3	1.246	16.5	11.4
1985**–**2012	8502	68.2	50.9	2.470	17.7	12.4

Numbers of cases and deaths, and age-standardized incidence and mortality rates (ASR-E and ASR-W) are shown for each period analyzed and for the first and last 5-year follow-up. Population: 463816 women residing in Granada province (Source: 2011 population census for Granada, Statistics and Cartography Institute of Andalusia). ASR-W: age-standardized rate referred to world population; ASR-E: age-standardized rate referred to European population. * per 100,000 women

**Fig. 1:**
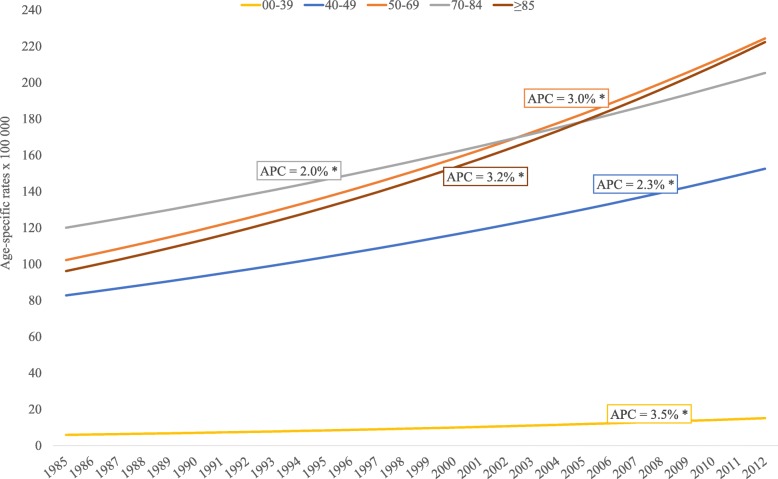
Age-specific incidence trends for breast cancer, 1985–2012. Joinpoint regression analysis of age-specific trends in breast cancer incidence rates per 100,000 for 1985–2012. APC estimates calculated by Joinpoint regression analysis. No change points were found. Population: 463816 women residing in Granada province (Source: 2011 population census for Granada, Statistics and Cartography Institute of Andalusia). * *p* < 0.05

Incidence trends by age group at diagnosis for the whole period showed an increase that was statistically significant in every age group, but differences were seen among groups (Fig. 1). A substantial proportion of cases (44.9%) were diagnosed in women 50**–**69 years old, and the APC was 3.0% (95%CI, 2.4–3.5). Age group 0–39 years accounted for 7.7% of all new cases, but presented the greatest increase (APC 3.5, 95%CI, 2.4–4.7). Positive trends were found for groups 40**–**49 years (APC 2.3, 95%CI, 1.5–3.0), 70–84 years (APC 2.0, 95%CI, 1.3–2.7), and 85 years and older (APC 3.2, 95%CI, 1.5–4.8) (Fig. 1).

### Incidence 2000–2012

**Fig. 2:**
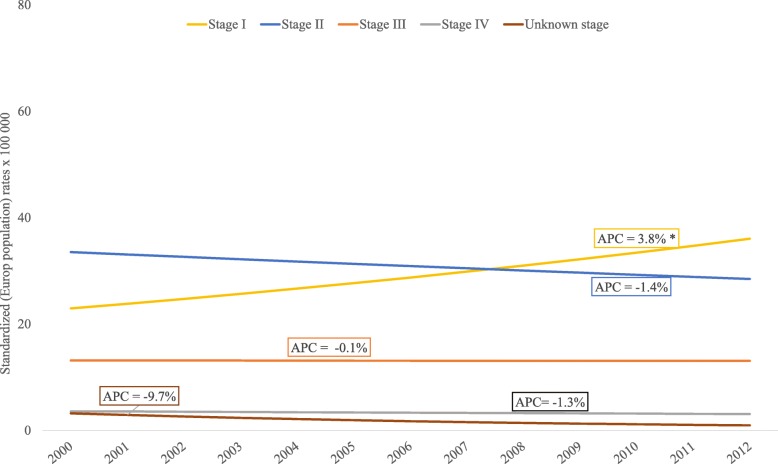
Age-standardized trends in breast cancer incidence according to tumor stage, 2000–2012. Joinpoint regression analysis of age-standardized trends in breast cancer incidence rates per 100,000 (referred to the European Standard Population) according to tumor stage at diagnosis for 2000–2012. APC estimates calculated by Joinpoint regression analysis. No change points were found. Age-standardized rates referred to the European population. Population: 463816 women residing in Granada province (Source: 2011 population census for Granada, Statistics and Cartography Institute of Andalusia). * *p* < 0.05

During this period 5120 new cases of breast cancer were registered, and incidence overall and for every age group showed a nonsignificant trend. An increase in incidence was found for stage I tumors (APC 3.8, 95%CI, 1.9–5.8), whereas a decrease was found for all other stages, although none of them reached statistical significance (Fig. 2). Distribution by stage showed that 35.0% of tumors were diagnosed in stage I, and 39.0% in stage II. Only 4.8% of all diagnoses were stage IV tumors. Distribution of breast cancer cases according to stage at diagnosis is shown by age group in Table 2 and by chronological year in Table 3.

**Table 2:** Tumor stage distribution by age group, 2000–2012

Age group (years)	I	II	III	IV	Unknown	Total
< 40	108 (29.1%)	172 (46.1%)	77 (20.6%)	12 (3.2%)	4 (1.1%)	373
40–49	360 (32.4%)	502 (45.2%)	192 (17.3%)	36 (3.3%)	21 (1.9%)	1111
50–69	991 (44.3%)	788 (35.3%)	312 (14.0%)	94 (4.2%)	49 (2.2%)	2234
70–84	296 (25.1%)	485 (41.2%)	264 (22.4%)	86 (7.3%)	47 (4.0%)	1178
≥85	31 (13.9%)	65 (29.2%)	70 (31.4%)	17 (7.6%)	40 (17.9%)	223
Overall	1792 (35.0%)	1998 (39.0%)	911 (17.8%)	243 (4.8%)	176 (3.4%)	5120

Number and percentage of breast cancer cases according to tumor stage and age group in 2000–2012. Percentages are rounded. Population: 463816 women residing in Granada province (Source: 2011 population census for Granada, Statistics and Cartography Institute of Andalusia)

**Table 3:** Tumor stage distribution by chronological year, 2000–2012

Year	I	II	III	IV	Unknown	Total
2000	104 (30.6%)	144 (42.4%)	54 (15.6%)	22 (6.5%)	17 (5.0%)	341
2001	102 (29.7%)	152 (44.3%)	64 (18.7%)	15 (4.4%)	10 (2.9%)	343
2002	111 (28.0%)	173 (43.6%)	71 (17.9%)	24 (6.0%)	18 (4.5%)	397
2003	94 (28.8%)	125 (38.4%)	70 (21.7%)	18 (5.6%)	18 (5.6%)	325
2004	115 (33.7%)	137 (39.9%)	62 (17.8%)	17 (4.4%)	14 (4.1%)	345
2005	119 (34.7%)	134 (39.1%)	57 (16.6%)	23 (6.7%)	10 (2.9%)	343
2006	127 (32.6%)	148 (38.0%)	87 (22.4%)	14 (3.6%)	13 (3.3%)	389
2007	133 (32.4%)	155 (37.8%)	85 (20.7%)	20 (4.9%)	17 (4.1%)	410
2008	154 (36.9%)	165 (39.8%)	70 (16.9%)	13 (3.1%)	14 (3.4%)	416
2009	204 (41.5%)	192 (39.1%)	69 (13.9%)	16 (3.1%)	12 (2.5%)	493
2010	174 (41.8%)	134 (32.2%)	70 (16.9%)	18 (4.5%)	19 (4.5%)	415
2011	175 (37.8%)	168 (36.6%)	77 (17.0%)	31 (6.9%)	8 (1.7%)	459
2012	180 (40.5%)	171 (38.3%)	75 (16.9%)	12 (2.9%)	6 (1.3%)	444
Overall	1792 (35.0%)	1998 (39.0%)	911 (17.8%)	243 (4.8%)	176 (3.4%)	5120

Number and percentage of breast cancer cases according to tumor stage and chronological year in 2000–2012. Percentages are rounded. Population: 463816 women residing in Granada province (Source: 2011 population census for Granada, Statistics and Cartography Institute of Andalusia)

### Mortality 1985–2012

The crude mortality rate for breast cancer during 1985–2012 in Granada province was 20.9 deaths per 100,000 women, corresponding to 2470 deaths. There was a decrease in ASR-E mortality from 20.5 to 15.2 per 100,000 women from 1985 to 1989 to 2008–2012 (Table 1). The mortality trend during the study period showed an annual decline (APC − 0.9, 95%CI, − 1.4 – − 0.5).

**Fig. 3:**
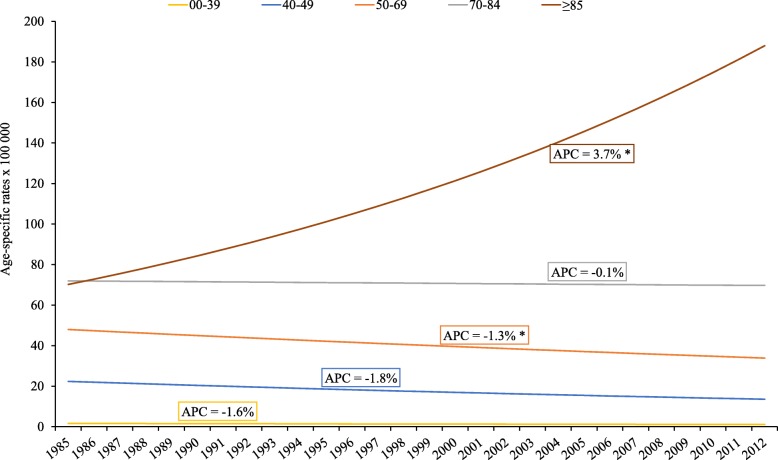
Age-specific mortality trends for breast cancer, 1985–2012. Joinpoint regression analysis of age-specific trends in breast cancer incidence rates per 100,000 for 1985–2012. APC estimates calculated by Joinpoint regression analysis. No change points were found. Population: 463816 women residing in Granada province (Source: 2011 population census for Granada, Statistics and Cartography Institute of Andalusia). * *p* < 0.05

Breast cancer deaths occurred mostly in women older than 70 years, and this age group contributed 45.3% of all deaths. However, only women older than 85 years showed an increasing trend in mortality (APC 3.7, 95%CI, 1.6–5.9) (Fig. 3). The rest of the age groups showed non-significant decreasing trends for this period, except the 50**–**69 year age group trend (APC − 1.3, 95%CI, − 2.2 – − 0.4).

### Mortality 2000–2012

The overall trend for this period showed a nonsignificant annual decrease of ˗0.7%. Stratification by age group showed a nonsignificant increasing trend in women aged 40**–**49 years (APC 4.2, 95%CI, − 1.8 – 10.4) and 85 years or more (APC 1.8, 95%CI, − 2.3 – 6.1). The number of deaths in these groups was 132 in the former and 180 in the latter (Table 4).

**Table 4:** Age-specific mortality trends for breast cancer in the female population in Granada province, 2000–2012

	N	APC	95%CI
Total	1246	−0.7	−2.3 – 0.9
Age group (years)			
00**–**39	35	−4.2	−12.0 – 4.2
40**–**49	132	4.2	−1.8 – 10.4
50**–**69	433	−2.7	−5.3 – 0.0
70**–**84	466	−1.2	−2.6 – 2.7
85 and over	180	1.8	−2.3 – 6.1

APC estimates calculated by Joinpoint regression analysis of age-specific mortality rates, for 2000–2012. Population: 463816 women residing in Granada province (Source: 2011 population census for Granada, Statistics and Cartography Institute of Andalusia). APC: annual percentage change

### Survival 1985–2012

**Fig. 4:**
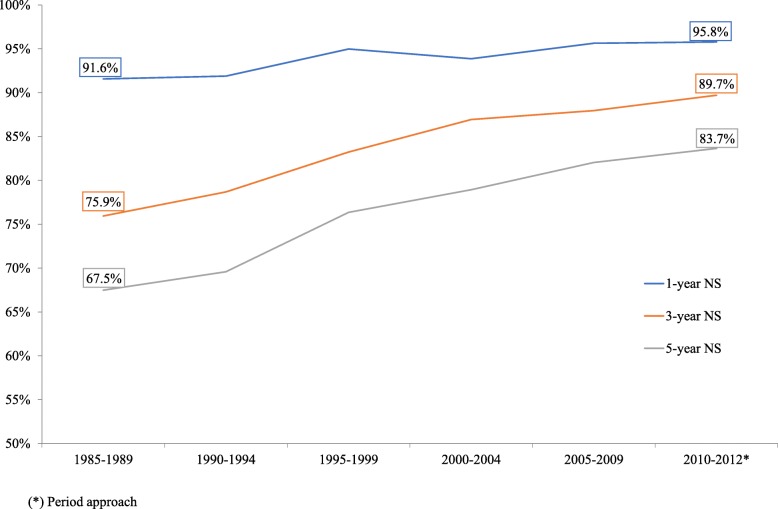
Age-standardized 1-, 3- and 5-year survival and net survival in women with breast cancer, 1985–2012. Estimates of observed survival calculated with the Kaplan–Meyer method, and net survival calculated with the Pohar–Perme method (cohort analysis) for 1985–2012 in 5-year periods and for the final 3-year period from 2000 to 2012. Period analysis was used instead of cohort analysis for the last 3-year period. Age-standardized rates referred to the European population. Population: 463816 women residing in Granada province (Source: 2011 population census for Granada, Statistics and Cartography Institute of Andalusia)

Both the observed and net age-standardized survival rates at 5 years increased steadily from 67.5% in 1985**–**1989 to 83.7% in 2010–2012 (Table 5). The evolution of survival rates 1, 3 and 5 years after diagnosis are illustrated in Fig. 4.

**Table 5:** Trends in observed 5-year survival and age-standardized net survival in women with breast cancer

Period	*n*	Observed survival			Net survival (age-standardized)		
		OS	95%CI		NS	95%CI	
1985**–**1989	844	63.9	60.5	67.0	67.5	61.8	72.5
1990**–**1994	1087	67.5	64.6	70.2	69.6	64.7	73.9
1995**–**1999	1344	73.7	71.3	76.0	76.4	72.4	79.8
2000**–**2004	1721	77.0	75.0	79.0	78.9	75.7	81.8
2005**–**2009	2030	80.0	78.2	81.7	82.1	79.0	84.7
2010**–**2012^a^	1791	81.0	78.8	83.1	83.7	79.8	86.8

^a^Period analysis instead of cohort analysis was used

Estimates for observed survival calculated with the Kaplan-Meyer method, and for net survival calculated with the Pohar–Perme method (cohort analysis) in 5-year periods from 1985 to 2012 and in the 3-year period from 2010 to 2012. Age-standardized rates referred to the European population. Population: 463816 women residing in Granada province (Source: 2011 population census for Granada, Statistics and Cartography Institute of Andalusia). OS: observed survival; NS: net survival

**Fig. 5:**
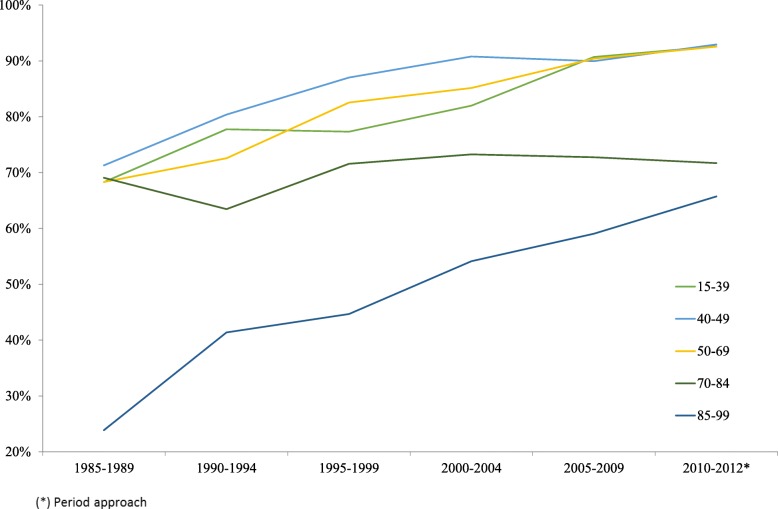
Five-year age-specific net survival in women with breast cancer, 1985–2012. Estimates of net survival calculated with the Pohar–Perme method (cohort analysis) for 1985–2012 in 5-year periods and for the final 3-year period from 2000 to 2012. Period analysis was used instead of cohort analysis for the last 3-year period. Population: 463816 women residing in Granada province (Source: 2011 population census for Granada, Statistics and Cartography Institute of Andalusia)

Age group analyses showed that survival tended to increase in groups younger than 70 years, with similar survival rates of approximately 90% in 2005**–**2009 and 93% in 2010**–**2012. For women 70 to 84 years old, survival increased to 70% until 1995**–**1999, and then remained stable (Fig. 5). The 85**–**99 year age group showed a constant increase in 5-year survival from approximately 20% in 1985**–**1989 to 60% in 2010**–**2012. This group accounted for the smallest number of deaths.

### Survival 2000–2012

Analysis by stage for the final twelve years of our study period disclosed important differences in survival related with disease progression from the moment of diagnosis. Net survival rates at 5 years were 96.6% in patients whose tumor was diagnosed in stage I, 88.2% for stage II tumors, and 62.5% for stage III tumors. The survival rate decreased markedly to 23.3% in women with stage IV tumors (Table 6).

**Table 6:** Age-standardized net survival according to stage in women with breast cancer, 2000–2012

Stage	1-year NS	3-year NS	5-year NS
I	99.9	98.3	96.6
II	99.7	95.1	88.2
III	91.4	75.3	62.5
IV	60.0	35.0	23.3
Unknown	92.3	77.4	75.4

Estimates for net survival calculated with the Pohar–Perme method (cohort analysis), according to tumor stage at the time of diagnosis (TNM 7th edition), 2000–2012

Age-standardized rates referred to the European population. Population: 463816 women residing in Granada province (Source: 2011 population census for Granada, Statistics and Cartography Institute of Andalusia). NS: net survival

## Discussion

The results we obtained here with data from the Granada Cancer Registry show a steady increase in breast cancer incidence between 1985 and 2012, with the greatest rise in women younger than 40 years and in the age group targeted for screening: 50**–**69 years. The decrease in breast cancer mortality and the upward trend in survival support a general improvement in prognosis. At the end of follow-up, women older than 84 years and those with metastatic spread at diagnosis were the groups showing the worst results.

From 1985 to 2012, the incidence of breast cancer in our population has increased, as documented by the APC of 2.5%. A similar increasing trend was observed in Europe, with APCs ranging between 0.8 and 3% [44]. The introduction of the screening program may have played an important role in this trend, as has been suggested in previous European studies [8, 10, 15–17, 36, 45, 46].

However, this trend started in our analysis before screening introduction and could also be found in age groups not invited to the program. These findings have been previously interpreted as indicators of the role played by environmental, lifestyle and behavioral exposures [3, 5, 8, 16, 23, 44, 45, 47–50]. Several breast cancer reproductive risk factors such as parity, advanced age at first birth or breast feeding have been highlighted before [51, 52], as well as lifestyle risk factors including alcohol consumption, post-menopausal obesity and sedentarism [53, 54]. Moreover, these finding have also been connected to changes in diagnostic practices, that have increased detection rates [10, 12, 16, 24, 45, 48], like the increasing use of opportunistic screening [55–57].

Finally, trying to disentangle the role played by screening program implementation in the increasing incidence from 1985 to 2012 shown in our population, we have performed a comparative analysis of overall and 50–69 year age group incidence trends before and after screening program introduction in our population in 1998. Incidence after 1998 showed a tendency to stabilization in overall analysis, even though trend showed a significant increase for both periods (1985–98 APC 2.9%, 1998–2012 APC 1.1%). Analysis of age group 50–69 years showed a positive, though non-significant trend after 1998 (1985–98 APC 2.8%, 1998–2012 APC 1.3%). These results suggest the influence of other determinants besides the screening program on the incidence trend shown from 1985 to 2012.

At the beginning of the twenty-first century, a change in this rising trend was seen in many European countries and in the USA. Screening programs initially led to a temporary increase in the incidence, followed by a decrease to pre-screening levels. This phenomenon is related to the diagnosis of silent prevalent cases in the first round and the need to wait until new incident cases occur in the screened population [44]. Moreover, a drop in breast cancer incidence correlated temporarily with the drastic reduction in menopausal hormone therapy in many countries [8, 15, 58] after the results of the WHI study were published [59]. These changes were observed in overall analyses and in postmenopausal women [8, 15]. However, none of these changes was found in our analysis of overall incidence, or in the 50**–**69 year age group.

A screening program in our population was introduced in 1998, and the whole target population was invited for the fifth round in 2002. In Granada the screening participation rate has been higher than 70% since 1999, and the median detection rate for the entire study period was 3.5‰. These surrogate indicators confirm the good performance of screening in our setting, according to the European Guidelines for Quality Assurance in Breast Cancer Screening and Diagnosis [60]. In light of this finding, the absence of changes in incidence trends seems not to be related to a reduced or delayed implementation of the program.

A previous study of the population analyzed here showed a temporary rise in incidence until 2004, similar to reports from other regions in Spain and consistent with the diagnosis of prevalent cases [9]. The longer follow-up period after screening introduction presented in our paper reduced the likelihood of finding smaller temporal trend changes in the Joinpoint analysis and could explain the absence of changes in incidence trend in our population. However, differences in age groups definition between both analyses, due to changes in the age range included in the Andalusian screening program, could also have played a role.

Some specific characteristics of our population may partially explain the absence of changes in temporal trends. During the study period, hormonal replacement therapy was prescribed to a lesser extent in Spain compared to other European countries [11, 61], so the increase in incidence during the 1990s and later decrease during the beginning of the 2000s due to the usual prescribing patterns were probably not as large as in other countries. Our setting (southern Spain) is at a relatively low socioeconomic level within the European Union, and this factor is known to be associated with a lower incidence [1]. This circumstance may mean a smaller number of silent prevalent cases at the beginning of the screening program, and hence a less dramatic fall after screening began. Finally, we should consider the effect of opportunistic screening as a source of potential bias, as previously described by international organisms [62]. This diagnostic practice shows high detection rates, especially for early stage and in-situ cancer [63], so it could have reduced the amount of prevalent cases that otherwise would have been detected in the first screening round [10]. No information is available regarding the extent of opportunistic screening in Granada province before or during our study period. However, this practice has been proved to be common in other countries [55], as well as in other regions of Spain before screening program introduction [56, 57], and its effect over incidence trends have been considered in previous studies [45, 64].

To better understand the effects of population-based screening, we undertook an analysis by tumor stage at the time of diagnosis for the period from 2000 to 2012. As expected, a statistically significant increase was observed in stage I tumors at diagnosis. The age distribution confirmed that this increase occurred mainly in the age group targeted for screening (50–69 years) – a trend consistent with earlier diagnosis due to screening. However, the absence of a parallel decrease in advanced-stage tumors in our distribution, has been attributed to the non-progressive nature of a large proportion of tumors potentially detectable by the program, and does not support this earlier diagnosis [17]. A favorable stage distribution due to screening is suggested by the lower proportion of stage III tumors in the screened age group (50**–**69 years old), but no decreasing trend was seen for this group in the Joinpoint analysis.

The decrease we observed in breast cancer mortality was noted throughout the whole period analyzed here. In Spain there has been a generalized decrease in mortality since 1992, although there is some variability among geographical regions [65]. This downward trend started in our cohort before the screening program was implemented, as in almost every region in Spain [65] and in other European countries [23]. Hormonal treatments and new polychemotherapy schemes were also introduced during the 1990s, and together with the increased use of effective radiotherapy regimens, probably played an important role in this trend [24, 36, 45, 66–68]. Metanalysis of the effectiveness of clinical trials with adjuvant treatments showed a marked reduction in breast cancer mortality, and in some cases, in all-cause mortality [31, 32].

The favorable evolution of survival trends is consistent with findings reported for other European countries [29] and the USA [27]; these trends correlate with tumor stage at diagnosis [69]. In our analysis, we found an increase in stage I tumors during the 2000–2012 period. Despite this favorable trend, survival did not increase in the 70**–**84 year group or in the subgroup with metastatic tumors at diagnosis. Adjuvant treatment, one of the factors responsible for this trend, is less effective for this stage and age group [70]. Women older than 70 years also have more comorbidities, and breast-conserving surgery plus adjuvant therapy are used to a lesser extent; both of these factors are related to decreased survival [71]. In the 85–99 year age group survival increased markedly from 23% in 1985**–**1989 to 62% in 2010**–**2012. However, the small number of deaths in this age group precludes any conclusions regarding this particular subgroup.

Mortality in women older than 70 years in Europe has shown an increasing trend or a smaller decrease than in younger age groups [19]. In our results, mortality increased in this age group (data not shown). This trend was also found for women older than 84 years in a separate analysis. In the 70**–**84 year and > 84 year age groups the proportion of metastatic tumors was larger than in other age groups (Table 2). Both older age and a greater proportion of metastatic tumors are important factors in the response to treatment. Moreover, women older than 70 years are less likely to receive standard treatment [72].

In our analysis of women younger than 40 years, the incidence trend (APC 3.6%) was larger than the trend reported for this age group in other European countries: the European median APC is 1.2% [13]. There appear to be no clear correlations between trends in this age group and known risk factors [13]. In younger women at least one earlier study found that factors related with tumor biology were associated with a greater risk of death and a worse prognosis [14].

Some authors have noted that changes in diagnostic patterns with the increased use of mammography and ultrasonography, along with wider access to MRI, are likely to be important factors in the reported increases in incidence among younger women [20]. In our study, more than 75% of tumors were diagnosed at stages I**–**II, and survival rates were similar to those in other age groups. These findings are consistent with the concurrent use of opportunistic screening in parallel with population-based screening programs. The influence of opportunistic screening was demonstrated in Barcelona, where 27.1% of women younger than 40 years received routine screening with mammography before a population-based program was introduced [57]. Moreover, 23.5% of women younger than 45 years reported having a mammography examination in 2014 [73], and 5% of this age group had visited a gynecologist for reasons other than pregnancy in the previous year [74]. Unfortunately, the lack of information regarding hormonal receptors and HER2 overexpression prevented us from analyzing these trends according to pathologic subtypes.

The decrease in overall mortality in Europe is reportedly greater in women younger than 50 years [19], and international studies confirm greater mortality with advancing age [5]. In our cohort, the 0**–**50 year age group showed a stable trend, in contrast to the decrease observed for women 50 to 69 years old (data not shown). In a differential analysis of the 40**–**49 year age group, we also found no statistically significant decrease. Previous research in Spain, however, reported a decrease in mortality among women younger than 40 years [75], so the difference between studies may reflect regional differences in incidence. However, caution should be used when interpreting these results given that the number of deaths in this age range is low.

The results of our analysis are strengthened by the inclusion of the most recent available data from the Granada population-based cancer registry, which has been in operation for 25 years and holds data for approximately 9000 registered cases of invasive breast cancer. We used appropriate statistical methods to detect trend changes and to calculated net survival rates. The high quality of the data was ensured by quality control measures, e.g. microscopic confirmation of the diagnosis in 96% of all registered cases, only 1.8% of which recorded the diagnosis based only on information from the death certificate.

Nevertheless, several limitations should be considered when interpreting our findings. The Granada Cancer Registry limits its target population to the provincial level, with a total population of approximately 1 million people. Population-specific characteristics need to be considered, along with the low number of events for some analyses. The absence of consistent information about risk factors prevalence in our population has not permitted us to present a direct interpretation of their role in time trends. Because of the mentioned factors, the external validity of our results may be limited, and the statistical power of some analyses was insufficient to reach definitive conclusions. In addition, lack of stage at diagnosis data before year 2000 refrained us from performing Joinpoint regression of breast cancer incidence according to stage for the whole period. Despite these limitations, this study provides population-based results for a long period of study, documents for the first time the evolution of breast cancer incidence according to stage at the time of diagnosis and provides some clues regarding the effect of screening on this important prognostic variable.

## Conclusions

In conclusion, the data from a population-based cancer registry in southern Spain show an increasing trend in breast cancer incidence from 1985 to 2012. The increases were greatest in the 0 to 49 and 50 to 69 years age groups. No change points in incidence trends were found in Joinpoint regression analysis for this period. This evolution is consistent with the spread of risk factors and the rise in diagnostic pressure [3, 8, 16, 23, 36, 44, 45, 47, 55, 76]. Further analyses of incidence trends determinants should be carried out, especially in young women, for designing future prevention strategies. We did not observe the previously turning point in incidence at the beginning of the twenty-first century in our country [8–10, 77] and, as we have already discussed, several causes may explain this difference.

Incidence trends by stage at diagnosis for the 2000**–**2012 period show an increase in stage I tumors. The absence of an equivalent decrease in advanced stage tumors suggests that at least a proportion of the tumors detected thanks to screening are non-progressive, raising doubts about screening effectiveness. However, the reduced proportion of stage III tumors in the 50**–**69 year age group points to a favorable shift in stage distribution. It should also be considered that tumors identified as metastatic at diagnosis represent a more aggressive type of breast cancer that may not benefit from mammographic screening, neither from advances in treatment. Therefore, it is possible that specific prevention strategies for metastatic breast cancer should be developed.

Mortality decreased slightly during the 1985**–**2012 period, although analysis by age group showed that this trend was statistically significant only in women aged 50**–**69 years. Although this is the age group the screening program is targeted to, the absence of any change in trend after screening was introduced, and the lack of a clear decrease in incidence during the 2000**–**2012 period, do not support a substantial beneficial effect.

The trend in survival of breast cancer in our setting has evolved favorably except in the 70**–**84 year age group, in which women more frequently receive non-standard treatments, and in which the percentage of stage I tumors – characterized by their better response to treatments – was lower than in other age groups.

## Abbreviations

APC: annual percentage change
ASR-E: age-standardized rate referred to European population
ASR-W: age-standardized rate referred to world population
CIFC: Cancer in Five Continents
ENCR: European Net of Cancer Registries
REDECAN: Spanish Network of Cancer Registries
WHI: Women Health Initiative
